# The ArcAB two-component system is associated with the susceptibility of *Aggregatibacter actinomycetemcomitans* to superoxide and hydrogen peroxide

**DOI:** 10.1128/msphere.00019-25

**Published:** 2025-04-16

**Authors:** Mohammad Farid Ratman, Yuichi Oogai, Airi Matsumoto, Masanobu Nakata

**Affiliations:** 1 Department of Oral Microbiology, Kagoshima University Graduate School of Medical and Dental Sciences 12851, Kagoshima, Japan; 2 Department of Oral and Maxillofacial Surgery, Kagoshima University Graduate School of Medical and Dental Sciences 12851, Kagoshima, Japan; The University of Iowa, Iowa City, Iowa, USA

**Keywords:** ArcAB, *Aggregatibacter actinomycetemcomitans*, superoxide dismutase

## Abstract

*Aggregatibacter actinomycetemcomitans* is a Gram-negative facultative anaerobe and is associated with periodontal disease. This bacterium is exposed to environmental stresses, such as osmotic pressure, temperature shifts, pH shifts, and antimicrobial substances, including reactive oxygen species (ROS), in the human oral cavity. The bacterial two-component system ArcAB modulates gene expression in response to environmental changes, primarily by sensing oxygen pressure in several pathogens belonging to the γ-proteobacteria. It is also known to provide adaptation to ROS stress; however, its function in *A. actinomycetemcomitans* remains unclear. In this study, we found that the expression of *sod*, which encodes superoxide dismutase, was increased in the inactivated mutant of *arcA*, which encodes a response regulator. The mutant exhibited reduced susceptibility to superoxide and hydrogen peroxide (H_2_O_2_). Additionally, this strain showed reduced susceptibility to H_2_O_2_ from *Streptococcus sanguinis* and increased survival in macrophages. Since ArcB is the cognate histidine kinase of ArcA, the inactivated mutant of *arcB* was analyzed for its phenotypes. The *arcB* mutant exhibited reduced susceptibility to superoxide and H_2_O_2_. Compared to wild type, the phosphorylation level of ArcA in the *arcB* mutant was decreased. These results suggest that the ArcA response regulator receives phosphate groups from ArcB histidine kinase and negatively regulates the expression of *sod*, thereby affecting bacterial survival in response to ROS produced by oral commensals and host immune cells.

**IMPORTANCE:**

*Aggregatibacter actinomycetemcomitans* is an oral pathogen that is known to be a highly virulent periodontal pathogen, showing strong adherence to periodontal tissue and toxin production, which leads to aggressive periodontitis. This bacterium is associated not only with oral infections but also with systemic infections, such as infective endocarditis and brain abscesses. Therefore, elucidating the adaptation mechanisms of this bacterium is important for human health. Bacterial two-component systems (TCSs) have been studied as attractive targets for elucidating bacterial fitness and pathogenicity in the host. This study characterized a TCS in *A. actinomycetemcomitans*, ArcAB, which is associated with susceptibility to ROS produced by host cells or oral commensals. Our findings provide insights into the bacterial adaptation mechanism against oxidative stress, which is crucial for understanding the survival strategies of the periodontal pathogen.

## INTRODUCTION

*Aggregatibacter actinomycetemcomitans* is a Gram-negative bacterium that is associated with periodontal disease, particularly aggressive forms of periodontitis (1). This organism is classified into seven serotypes (serotypes a, b, c, d, e, f, and g) based on the antigenicity of the O-antigen of lipopolysaccharide (2–5). Serotype b strains are associated with aggressive periodontitis (6). Serotypes a–c are globally dominant, whereas serotypes d–f are relatively rare in periodontitis (7). The newly identified serotype g strain has been detected in chronic periodontitis and is associated with strong biofilm formation (8).

*A. actinomycetemcomitans* evades host immune responses and induces inflammation and destruction of periodontal tissues using several virulence factors, such as exotoxins, lipopolysaccharides, outer membrane proteins, and outer membrane vesicles (9–13). In the oral cavity, bacteria are exposed to a variety of environmental stresses, such as osmotic pressure, temperature shifts, pH shifts, and antimicrobial factors that are produced by oral commensals and host cells. Among these antimicrobial factors, reactive oxygen species (ROS), including superoxide anion radical (O_2_^•−^), hydrogen peroxide (H_2_O_2_), and hydroxyl radical (OH^•^), exert antimicrobial activity through the oxidation of nucleic acids, proteins, and lipids (14–16). To cope with ROS stress, *A. actinomycetemcomitans* possesses protective mechanisms, such as the production of antioxidant enzymes (17). Catalase (*katA*) provides protection against exogenous H_2_O_2_ and H_2_O_2_ produced by *Streptococcus gordonii* in *A. actinomycetemcomitans* and *Aggregatibacter aphrophilus* (18). Superoxide dismutase (SOD) expressed in the periplasm provides protection against O_2_^•−^ (19). Quinol peroxidase contributes to aerobic growth and the scavenging of exogenous H_2_O_2_ (20). In many bacterial species, these antioxidant genes are regulated by several regulatory factors, such as PerR, OxyR, SoxR, and two-component systems (TCSs) (21–24). However, the regulation of these antioxidant genes in *A. actinomycetemcomitans* remains unknown.

TCSs are crucial for adaptation to environmental stress (25, 26), and typically consist of a membrane-bound histidine kinase (HK) and a response regulator (RR), whose genes are organized within an operon. When an HK senses a specific signal from the environment and autophosphorylates, its corresponding RR receives a phosphate group from the HK, and the phosphorylated RR often acts as a transcriptional regulator of the target genes (27). In *A. actinomycetemcomitans*, QseBC is the most extensively studied TCS and is known to influence biofilm formation (28) and bone loss in periodontal tissue (29). QseBC is activated by catecholamine hormones and iron (30) as well as by epinephrine stored in azurophilic granules of human neutrophils (31).

ArcAB is found in certain γ-proteobacteria, such as Alteromonadaceae, Enterobacteriaceae, Vibrionaceae, and Pasteurellaceae. Several studies on the functional role of ArcAB have been performed using various pathogens, such as *Escherichia coli*, *Salmonella enterica* serovar Typhimurium, *Vibrio cholerae*, and *A. actinomycetemcomitans* (26, 32–34). ArcAB in *E. coli* primarily contributes to the regulation of oxygen availability, anaerobic respiration, and energy metabolism (35, 36). In facultative anaerobes, regulation via ArcAB is needed for the transition from aerobic respiration to fermentation or anaerobic respiration when the amount of available oxygen is limited (35–38).

Several studies have shown that the ArcAB system acts as a global regulator not only for bacterial respiration but also for ROS resistance and virulence (32, 39–41). In *E. coli*, *arcA* inactivation did not alter the H_2_O_2_ scavenging ability but led to overproduction of flagellin, which resulted in reduced susceptibility to H_2_O_2_ through the depletion of resources for protein synthesis in mutant cells (32). Additionally, *S*. Typhimurium represses porin expression through ArcAB and controls H_2_O_2_ uptake (39). *Salmonella enterica* serovar Enteritidis uses ArcAB to control resistance to reactive nitrogen species and H_2_O_2_ (40). In *A. actinomycetemcomitans*, Longo et al*.* demonstrated that *arcB* inactivation attenuated biofilm formation and adhesion to saliva-coated surfaces (34).

The ArcAB TCS has been recognized as an atypical TCS, as ArcA and ArcB are not encoded by genes in a single operon (42), and ArcB is missing in many species of Alteromonadaceae (43). In this study, we analyzed genes regulated by the ArcAB system in *A. actinomycetemcomitans* and investigated their effects on bacterial susceptibility to superoxide and H_2_O_2_ as well as bacterial survival in macrophages.

## RESULTS

### Gene expression of strains lacking *arcA* or *arcB*

**Fig 1:**
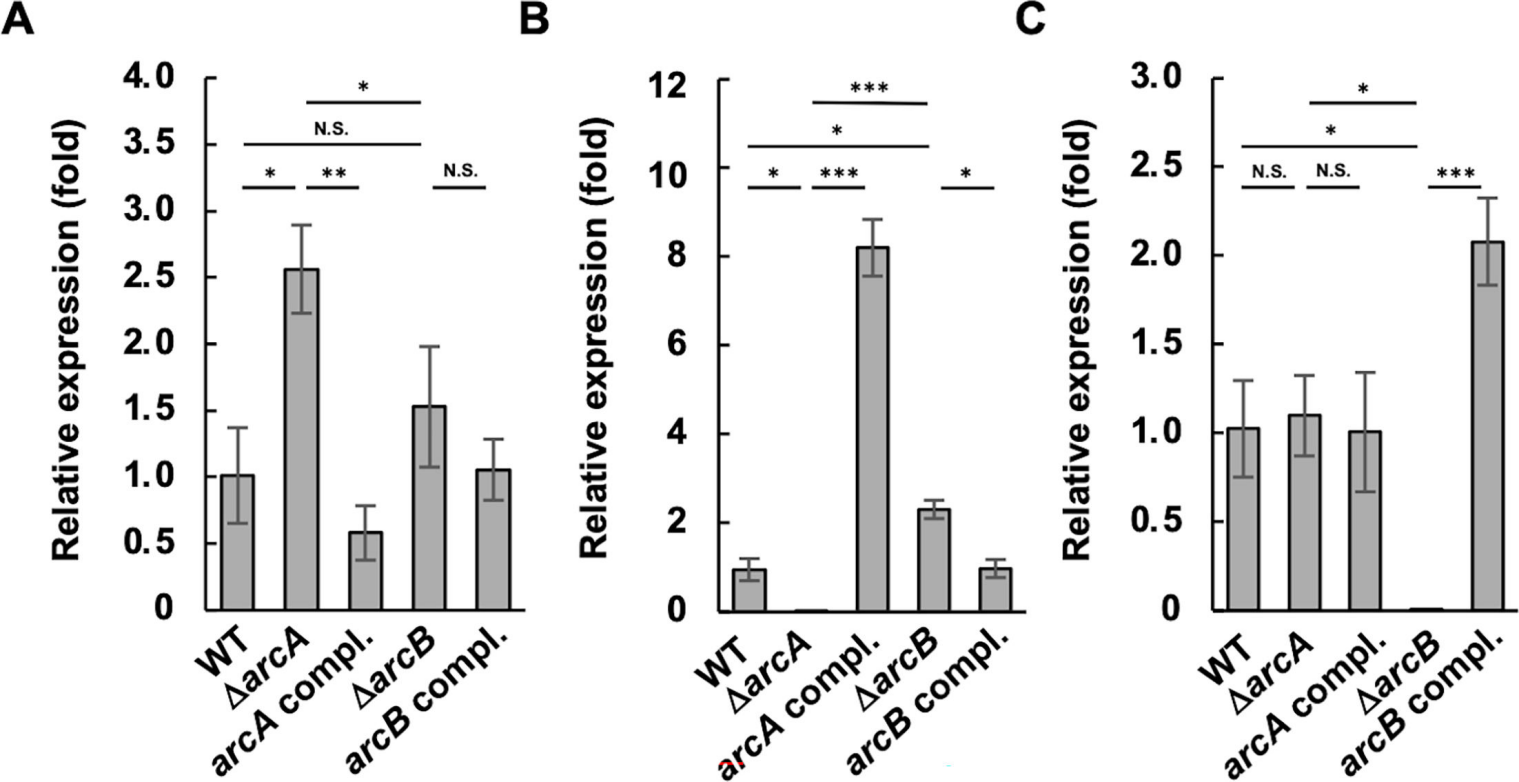
Gene expression analysis in *A. actinomycetemcomitans* strains. The gene expression levels of the *A. actinomycetemcomitans* NUM4039 wild-type (WT) strain, *arcA* and *arcB* mutants (*ΔarcA* and *ΔarcB*), and their respective complemented strains (*arcA* compl. and *arcB* compl.) were analyzed by qRT-PCR. The expression levels of *sod* (**A**), *arcA* (**B**), and *arcB* (**C**) were determined in cells grown to the mid-exponential phase (OD_660_ = 0.6), with *gapdh* serving as an internal control. The relative expression (fold) was determined using the expression level in WT as the calibrator. The data represent the means ± SDs from three independent experiments, each with three biological replicates. Statistical significance was determined by Tukey’s test (*P < 0.05; **P < 0.001; ***P < 0.001; and N.S., not significant).

*A. actinomycetemcomitans* NUM4039 wild-type (WT) strain, its isogenic *arcA* and *arcB* mutants (Δ*arcA* and Δ*arcB*), and their respective complemented strains (*arcA* compl. and *arcB* compl.) were cultured in *A. actinomycetemcomitans* growth medium (AAGM) (44) under ambient air supplemented with 5% CO_2_. The growth kinetics of the WT and the mutants were almost similar (Fig. S1). To identify genes regulated by the ArcA RR in *A. actinomycetemcomitans*, differentially expressed genes were analyzed by RNA sequencing (RNA-seq) in the mid-exponential phase (optical density at 660 nm [OD_660_] reached 0.6) cells of NUM4039 WT and Δ*arcA* cultured in AAGM under ambient air supplemented with 5% CO_2_. The result of the RNA-seq analysis was visualized in an MA plot (Fig. S2). After removal of reads showing the fragments per kilobase of transcript per million fragments mapped (FPKM) value below 10, the data revealed that 68 genes were differentially expressed, with 40 genes upregulated more than twofold and 28 genes downregulated more than twofold (Table S1 and S2). Interestingly, the expression of the *sod* gene, which encodes SOD, was increased 2.84-fold in the Δ*arcA* compared to the WT (Table S1). The increase in *sod* expression in Δ*arcA* was confirmed by quantitative reverse transcription PCR (qRT-PCR) (Fig. 1A). The increased expression of *sod* in Δ*arcA* was also observed at the early exponential and stationary phases, and in a bacteriostatic state induced by iron depletion (Fig. S3). The *arcA* compl. showed reduced *sod* expression compared to Δ*arcA* (Fig. 1A; Fig. S3). The strain lacking *arcB* (Δ*arcB*), which encodes a HK, did not show a statistically significant increase in *sod* expression (Fig. 1A; Fig. S3). The expression of *arcA* was increased 2.31-fold in Δ*arcB* compared to the WT (Fig. 1B). The *arcA* compl. showed an 8.2-fold increase in *arcA* expression compared to the WT. The complemented strain contains the entire *arcA* sequence with its native promoter in the multicloning site of the pJAK16 plasmid (45), which has an isopropyl β-d-1-thiogalactopyranoside (IPTG)-inducible P*_tac_* promoter located upstream of the multiple cloning sites. Since we realized that the P*_tac_* promoter was leaky in *A. actinomycetemcomitans* during preliminary tests, the strains were cultured in media without IPTG. However, the *arcA* expression level was significantly greater in the complemented strain than in the WT strain. The expression of *arcB* was unchanged in Δ*arcA* and increased in the *arcB* compl. (Fig. 1C).

### Susceptibility of Δ*arcA*, Δ*arcB*, and Δ*sod* to O_2_^•−^

**Fig 2:**
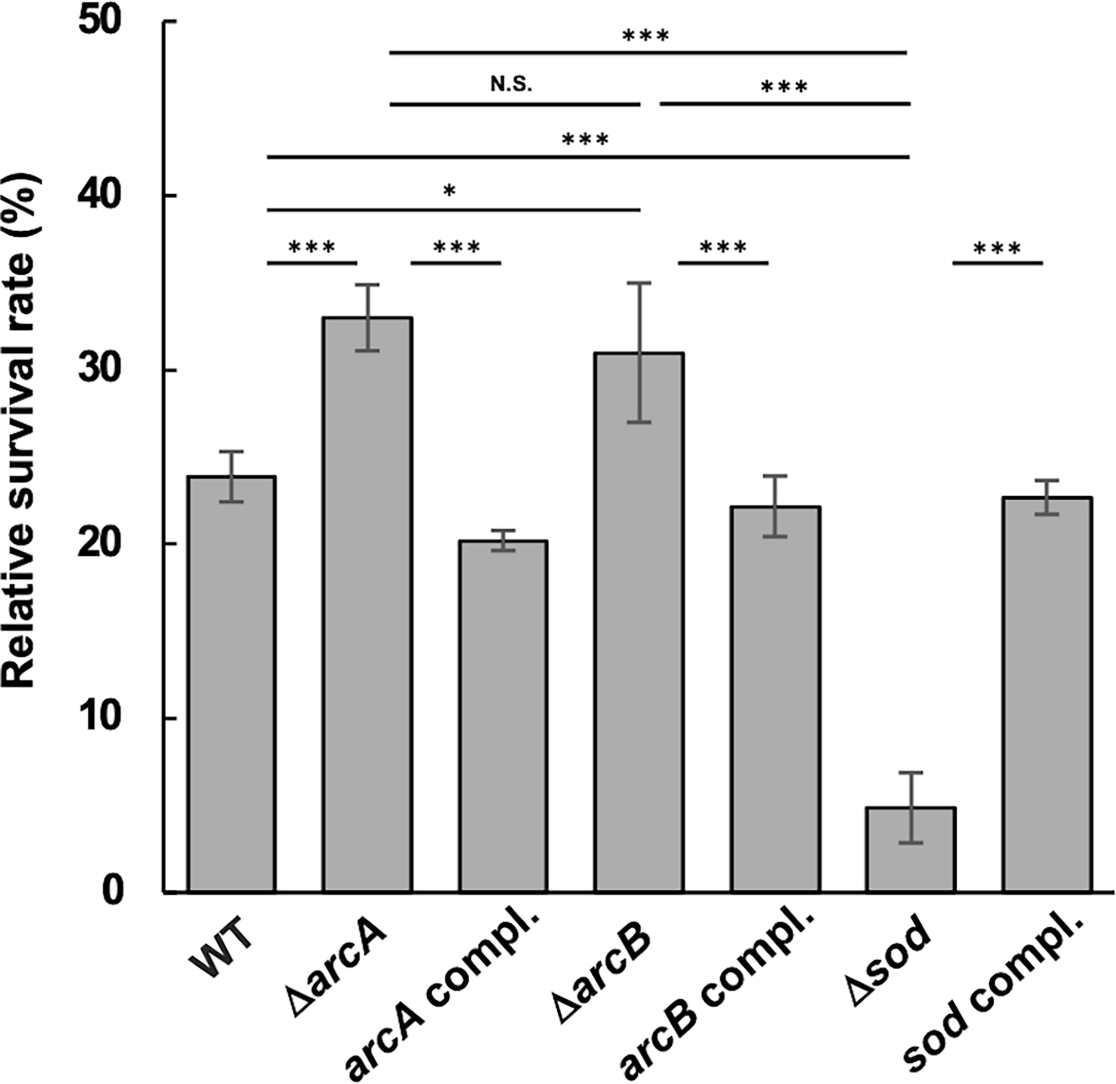
Susceptibility of *A. actinomycetemcomitans* strains to superoxide anion radical (O_2_^•−^). The *A. actinomycetemcomitans* strain NUM4039 and its isogenic mutants were grown to the mid-exponential phase (OD_660_ = 0.6) and exposed to O_2_^•−^ generated by the xanthine-xanthine oxidase reaction at 37°C for 30 min. The cells were then plated on agar plates to determine their survival rates. Colony counts from untreated controls were set as 100%. The data represent the means ± SDs from three independent experiments, each with three biological replicates. Statistical significance was determined by Tukey’s test (*P < 0.05; **P < 0.001; ***P < 0.001; and N.S., not significant).

Since Δ*arcA* presented increased *sod* expression, the susceptibility of the WT and mutant strains to O_2_^•−^ was tested. To analyze the function of *sod* in *A. actinomycetemcomitans*, a *sod* mutant (Δ*sod*) was also constructed and tested. SOD is an antioxidant enzyme that converts O_2_^•−^ into H_2_O_2_ (46, 47). The survival rates of all the strains were lower than those in the absence of O_2_^•−^, with the most remarkable reduction observed in Δ*sod* (Fig. 2). In contrast, the survival rates of Δ*arcA* and Δ*arcB* were significantly higher than that of the WT. The survival rates of the respective complemented strains of Δ*arcA*, Δ*arcB*, and Δ*sod* (*arcA* compl., *arcB* compl., and *sod* compl., respectively) were restored to WT levels. These data indicate that the increased expression of *sod* in Δ*arcA* and Δ*arcB* correlates with the susceptibility of these strains to O_2_^•−^.

### Susceptibility of Δ*arcA*, Δ*arcB*, and Δ*sod* to H_2_O_2_

In the oral cavity, *A. actinomycetemcomitans* coexists with commensal bacteria, such as H_2_O_2_-producing *Streptococcus sanguinis* (48). Since a *sod* mutant was shown to be highly susceptible to H_2_O_2_ in a previous study using *Streptococcus mutans* (49), the susceptibilities of Δ*arcA*, Δ*arcB*, and Δ*sod* to H_2_O_2_ were analyzed. Two types of susceptibility tests were conducted.

**Fig 3:**
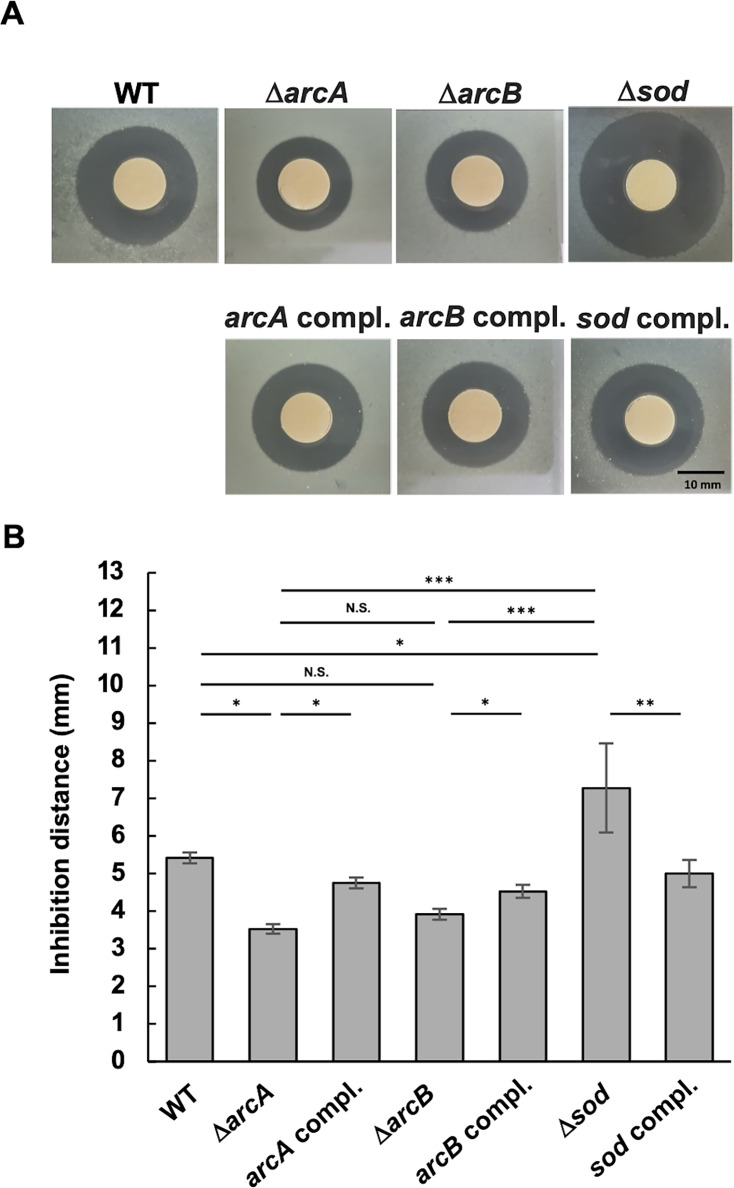
Susceptibility of *A. actinomycetemcomitans* strains to H_2_O_2_. (**A**) The *A. actinomycetemcomitans* strain NUM4039 and its isogenic mutants were grown to the mid-exponential phase (OD_660_ = 0.6), suspended in soft TSB-YE agar (0.75% agar), and overlaid on TSB-YE agar plates. Sterile paper discs containing 10 µL of 3% H_2_O_2_ were placed on the agar surface, and the plates were incubated at 37°C for 2 days. Representative images show growth inhibition zones around the discs. Scale bar, 10 mm. Five independent experiments were performed. (**B**) Growth inhibition was quantified by measuring the distance from the edges of the inhibition zone to the edges of a paper disc in three directions. The data represent the means ± SDs from five independent experiments. Statistical significance was determined by Tukey’s test (*P < 0.05; **P < 0.001; ***P < 0.001; and N.S., not significant).

First, *A. actinomycetemcomitans* strains in a half-strength agar medium were exposed to 3% H_2_O_2_ on a filter disk (Fig. 3A and B). Compared to the WT, the growth inhibition zones for Δ*arcA* and Δ*arcB* were smaller, whereas those for Δ*sod* were larger (Fig. 3A). There were no significant differences in zone size between the WT and complemented strains. The degree of susceptibility to H_2_O_2_ was evaluated by measuring the distance between the distal edge of the disk and the growth inhibition zone (Fig. 3B). On average, the distances for Δ*arcA* and Δ*arcB* were 1.9 and 1.5 mm shorter, respectively, than those for the WT. However, Δ*arcB* showed no statistically significant difference compared to the WT. These data indicated that Δ*arcA* was less susceptible to H_2_O_2_, whereas Δ*sod* was more susceptible.

**Fig 4:**
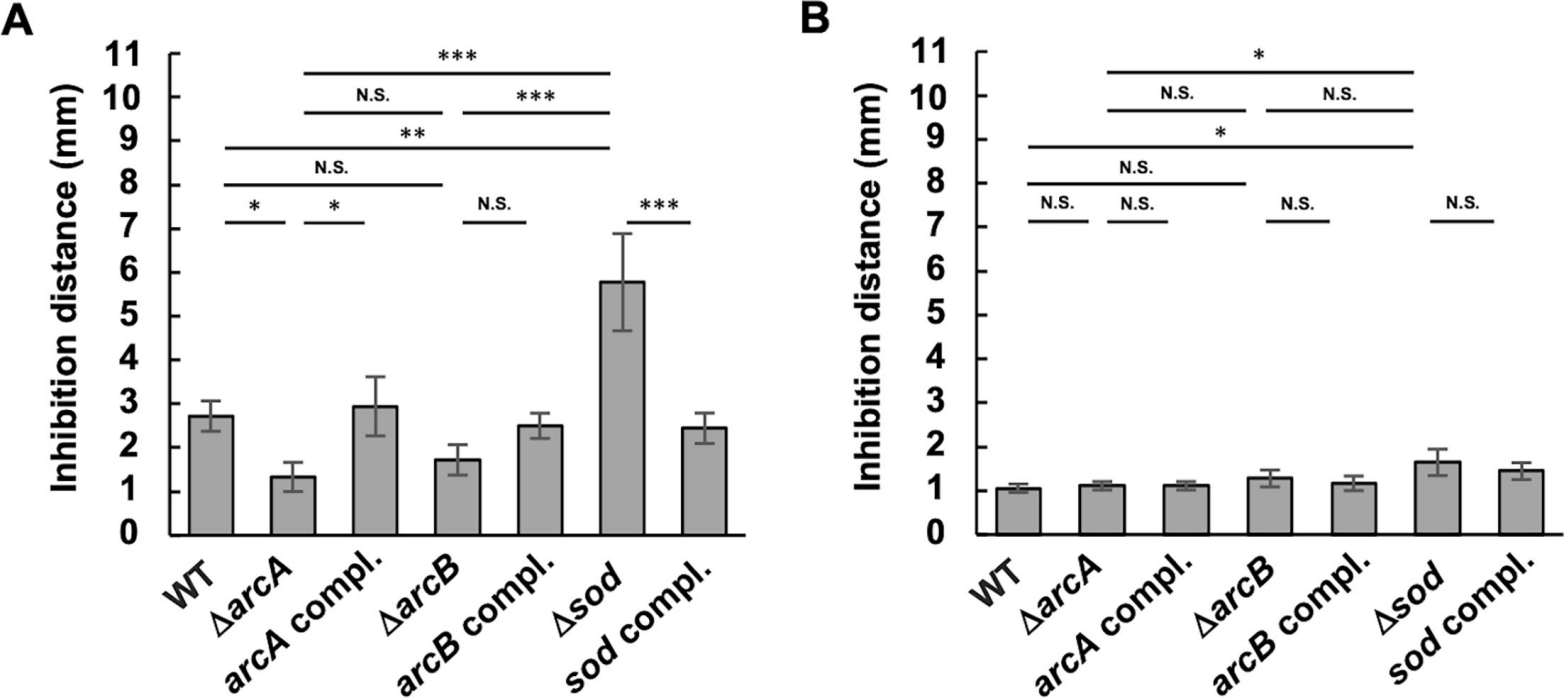
Growth inhibition of *A. actinomycetemcomitans* strains by H_2_O_2_-producing *S. sanguinis*. The *A. actinomycetemcomitans* strain NUM4039 and its isogenic mutants grown to the mid-exponential phase were suspended in soft TSB-YE agar (0.75% agar) and overlaid on TSB agar plates containing a colony of either *S. sanguinis* strain SK36 (**A**) or its isogenic *spxB* mutant lacking pyruvate oxidase (**B**). The plates were incubated at 37°C for 2 days. Growth inhibition was quantified by measuring the distance from the edges of the inhibition zone to the edges of *S. sanguinis* colonies in three directions. The data represent the means ± SDs of three independent experiments. Statistical significance was determined by Tukey’s test (*P < 0.05; **P < 0.001; ***P < 0.001; and N.S., not significant).

Next, *A. actinomycetemcomitans* strains in half-strength agar media were layered over an *S. sanguinis* colony grown on a trypticase soy broth (TSB) agar plate. After incubation, the growth inhibition zones of the *A. actinomycetemcomitans* strains were examined by measuring the distance between the distal edge of the *S. sanguinis* colony and the growth inhibition zone. For this assay, we employed previously constructed strains: the *S. sanguinis* strain SK36 (50) and its isogenic mutant lacking *spxB* (Δ*spxB*), a gene encoding pyruvate oxidase required for H_2_O_2_ production (51). Coculture with SK36 resulted in reduced inhibition zones for Δ*arcA* but increased zones for Δ*sod* compared to the WT (Fig. 4A). ∆*arcB* tended to show reduced inhibition zones, but with no statistical difference. The inhibition zones of the complemented strains were similar to those of the WT. To confirm whether growth inhibition was attributable to H_2_O_2_ produced by SK36, Δ*spxB* was subjected to the same assay. All *A. actinomycetemcomitans* strains presented small inhibition zones (<2 mm) around colonies of Δ*spxB* (Fig. 4B), suggesting that the *arcA* and *sod* mutations altered the susceptibility to H_2_O_2_ produced by *S. sanguinis*.

### Bacterial survival of Δ*arcA*, Δ*arcB*, and Δ*sod* in THP-1 macrophages

**Fig 5:**
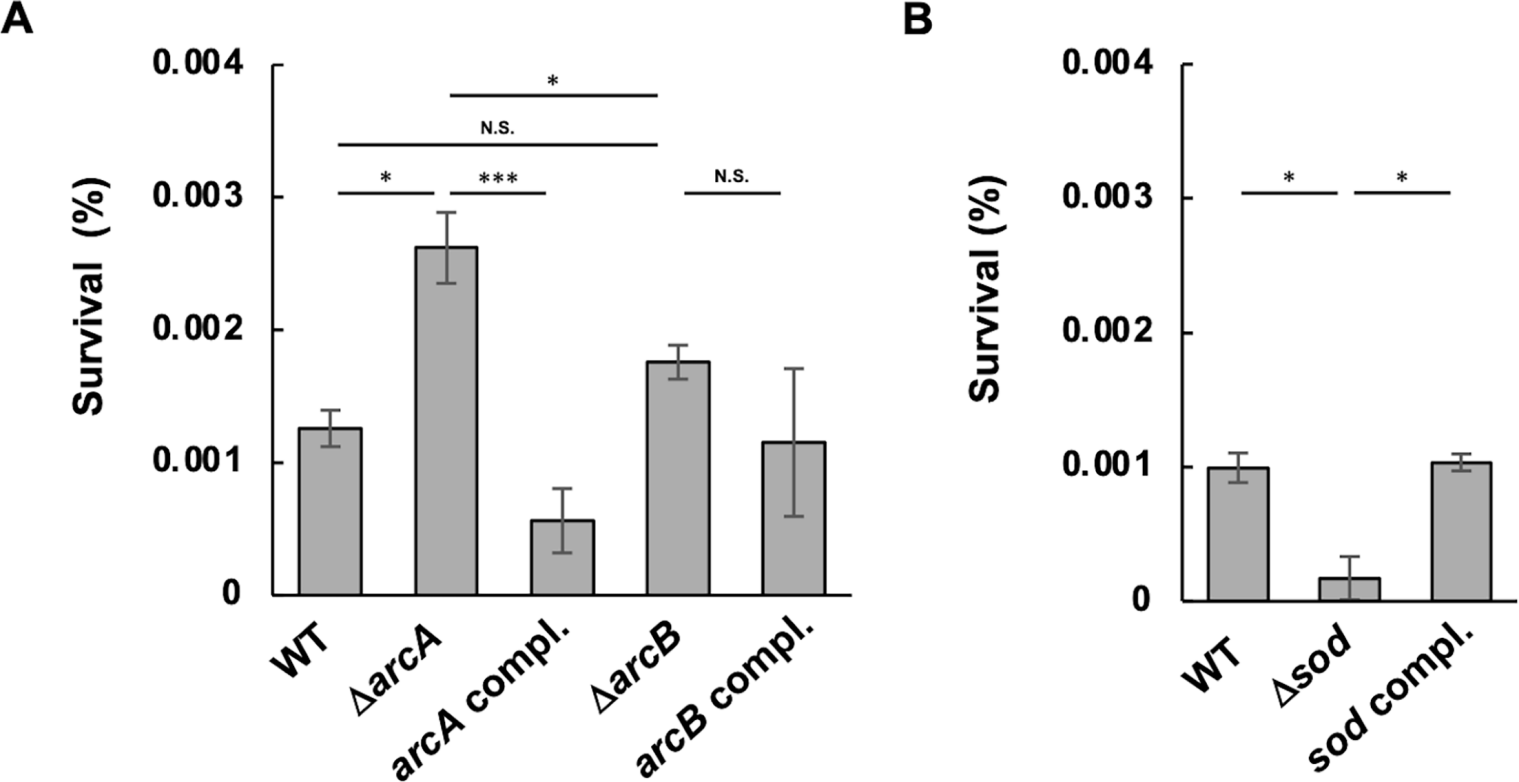
Survival of *A. actinomycetemcomitans* strains in THP-1 macrophages. THP-1 macrophages were infected with the *A. actinomycetemcomitans* strain NUM4039 and its isogenic mutants at a multiplicity of infection (MOI) of 50 and incubated at 37°C for 90 min. After the bacteria were killed with gentamicin, the macrophages were lysed, and the intracellular bacteria were enumerated. Survival rates are expressed as percentages of the initial inoculum. The data represent the means ± SDs from three independent experiments, each with three biological replicates. Statistical significance was determined by Tukey’s test (*P < 0.05; ***P < 0.001; and N.S., not significant).

Since ROS contribute to bactericidal activity in phagocytes and the ArcAB system influences the susceptibility of *A. actinomycetemcomitans* to O_2_^•−^ and H_2_O_2_, we tested whether the ArcAB system affects bacterial survival in THP-1 macrophages. THP-1 cells were differentiated into macrophages and infected with *A. actinomycetemcomitans* strains. After the elimination of the extracellular bacteria, the surviving bacteria were recovered from the cell lysates. Compared to the WT strain, Δ*arcA* showed an increased survival rate, and the *arcA* complemented strain presented similar survival rates, while Δ*arcB* showed no statistically significant difference compared to the WT (Fig. 5A). In addition, Δ*sod* showed a lower survival rate compared to the WT, and the complemented strain presented a rate similar to that of the WT strain (Fig. 5B). Hence, ArcA-mediated regulation of *sod* affects *A. actinomycetemcomitans* survival within macrophages.

### Evaluation of ArcA phosphorylation via ArcB

**Fig 6:**
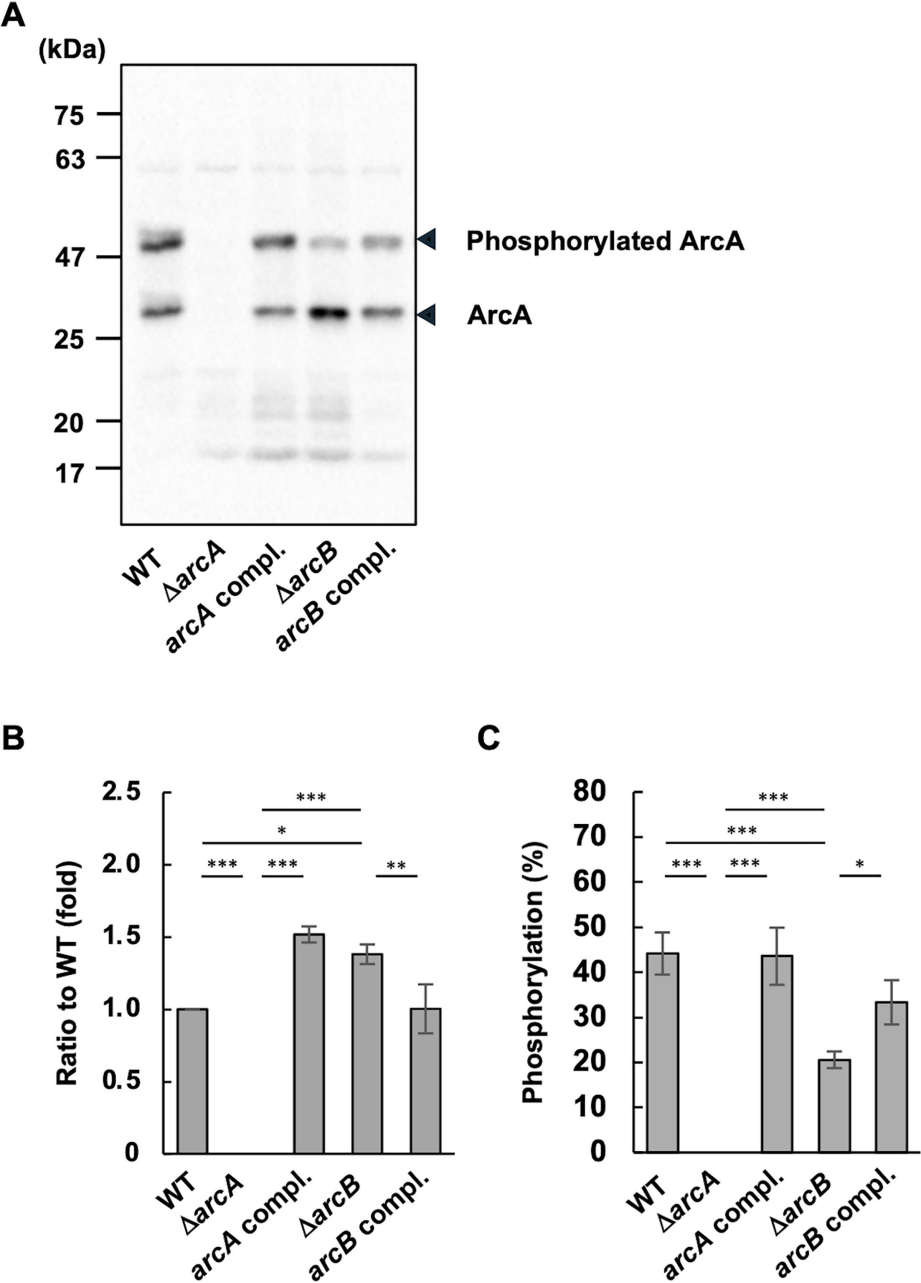
Analysis of ArcA phosphorylation in *A. actinomycetemcomitans* strains. Total proteins in homogenates of the *A. actinomycetemcomitans* strain NUM4039 and its isogenic mutants grown to the mid-exponential phase were sedimented on 12.5% Phos-tag gels to resolve the phosphorylated and non-phosphorylated forms of ArcA. Immunoblot analyses were conducted with rabbit anti-ArcA antiserum and horseradish peroxidase-conjugated anti-rabbit IgG antibody. (A) Representative immunoblot data from five independent experiments. (B) Total ArcA levels in each strain relative to those in the WT strain were quantified by densitometry using standard SDS-PAGE. (C) The ratio of phosphorylated ArcA to total ArcA was determined using Phos-tag gels. The data represent the means ± SDs from five independent experiments. Statistical significance was determined by Tukey’s test (*P < 0.05, **P < 0.001, and ***P < 0.001).

To examine the levels of phosphorylated ArcA, we used a Phos-tag gel to distinguish between the phosphorylated and non-phosphorylated forms of ArcA. Immunoblot analysis was performed using cell lysates from the WT, Δ*arcA*, Δ*arcB*, and complemented strains. In the WT lysates, we detected two signals of approximately 48 and 27.5 kDa, representing phosphorylated and non-phosphorylated ArcA, respectively (Fig. 6A). Neither signal was detected in Δ*arcA* lysates, which served as a negative control (Fig. 6A). Notably, in Δ*arcB* lysate, the level of phosphorylated ArcA was markedly decreased, whereas the level of non-phosphorylated ArcA was increased.

Using data from five independent experiments, we performed densitometric analyses (Fig. 6B and C). ArcA signals obtained using standard gels revealed increased ArcA levels in Δ*arcB* compared to the WT, which was consistent with the qRT-PCR data (Fig. 1B and 6B). The ratio of phosphorylated ArcA to total ArcA was significantly decreased in Δ*arcB* and was partially restored by complementation (Fig. 6C). Although the total ArcA levels increased in the complemented strain of Δ*arcA* (Fig. 6B), the ratio of phosphorylated ArcA remained unchanged (Fig. 6C).

The immunoblot assay was also performed using lysates from the IDH781 strain and its isogenic mutants (Table S3). A decrease in phosphorylated ArcA levels in Δ*arcB* was also clearly observed (Fig. S4). Therefore, ArcB likely phosphorylates ArcA. However, since phosphorylated ArcA signals remained in Δ*arcB* lysates, we investigated whether other TCSs, including CpxAR and PgtABC, were involved in ArcA phosphorylation. We constructed TCS deletion mutants (Δ*cpxAR* and Δ*pgtABC*) in the IDH781 background and performed immunoblot analysis. The ArcA phosphorylation levels did not significantly decrease (Fig. S4), suggesting the involvement of other factors.

## DISCUSSION

ArcAB has been reported to modulate the expression of genes involved in carbon source oxidation, fermentation, and nitrogen metabolism. The ArcAB system in *E. coli* and *Salmonella* species has been extensively studied as a key regulator that controls the switch from aerobic respiration to fermentation or anaerobic respiration under oxygen-limited conditions (52–56). In the present study, we analyzed the transcriptome of an *A. actinomycetemcomitans* ∆*arcA* mutant under aerobic conditions (5% CO_2_) and found that genes involved in aerobic carbon flow (*aceEF*, *lpdA*, *sucCD*, and *lldD*) were upregulated in ∆*arcA* (Fig. S2 and Table S1). Among the genes upregulated in ∆*arcA*, *fdnG*, *fdxH*, and AANUM_RS10625 were the most highly upregulated and may form an operon. These genes correspond to the *E. coli fdnGHI* operon, which encodes subunits of formate dehydrogenase N (57). In a study using *H. influenzae*, *fdxH* and *fdxI*, which correspond to *fdxH* and AANUM_RS10625 in *A. actinomycetemcomitans*, respectively, were listed as upregulated genes in ∆*arcA* (41). These findings suggest that ArcA modulates the expression of genes involved in carbon source metabolism and formate dehydrogenase in *A. actinomycetemcomitans*. However, the Δ*arcA* mutant did not show altered growth kinetics under our experimental conditions (Fig. S1).

Our RNA-seq data revealed that the most downregulated genes were from the *nrdDG* operon (Fig. S2 and Table S2), which is responsible for DNA synthesis under anaerobic conditions in *E. coli* (58). In *E. coli*, the expression of *nrdDG* is regulated by the Fnr (fumarate and nitrate reduction) protein, a global regulator of oxygen utilization (59), but not by ArcAB (60). Comparative genomic analyses have suggested that Fnr has lost its role as a main regulator in Pasteurellaceae compared to its role in Enterobacteriaceae and Vibrionaceae (61). These previous reports, together with our findings, suggest that *nrdDG* expression may be controlled by ArcAB in Pasteurellaceae.

In the present study, we found that *sod* expression was increased in Δ*arcA*, whereas its susceptibility to O_2_^•−^ and H_2_O_2_ was decreased (Table S1; Fig. 1 through 4). We also confirmed that Δ*sod* was highly susceptible to O_2_^•−^ in *A. actinomycetemcomitans* (Fig. 2), which is consistent with previous findings from *A. actinomycetemcomitans* strain JP2, in which the Δ*sod* mutant presented increased susceptibility to O_2_^•−^ (19). Furthermore, *A. actinomycetemcomitans* susceptibility to H_2_O_2_ was decreased by inactivation of *arcA* (Fig. 3). Since the most harmful ROS, OH^•^, can be generated from accumulated endogenous O_2_^•−^ reacting with exogenous H_2_O_2_ (O_2_^•−^ + H_2_O_2_ → O_2_ + OH^−^ + OH^•^), increased *sod* expression in Δ*arcA* may reduce endogenous O_2_^•−^ levels, potentially increasing cell survival in the presence of exogenous H_2_O_2_. However, the reduced H_2_O_2_ susceptibility in Δ*arcA* conflicts with the findings of previous studies on other bacterial species. In *E. coli,* inactivation of *arcA* and *arcB* increased susceptibility to H_2_O_2_ (32). Wong et al*.* demonstrated that *arcA* inactivation in *H. influenzae* significantly reduced survival rates in the presence of H_2_O_2_ under anaerobic conditions and decreased the expression of *dps*, which encodes an inhibitory protein against OH^•^ generation, in the *arcA* mutant (41). The *dps* gene is present in the *A. actinomycetemcomitans* NUM4039 genome (AANUM_RS06660) but has been identified as a pseudogene. Additionally, the expression of other antioxidant factor genes, such as *katA* (catalase) and *qpo* (quinol oxidase), was unchanged in Δ*arcA* (data not shown). These data suggest that the negative regulation of *sod* by ArcA is characteristic of *A. actinomycetemcomitans* and that the reduced H_2_O_2_ susceptibility in Δ*arcA* is attributed to increased *sod* expression rather than changes in *katA* and *qpo* expression.

In the human body, the nutrient composition differs significantly from that of bacterial culture media, with free iron strictly limited (62). Therefore, it is important to confirm the regulation of *sod* by ArcA in iron-depleted cells, which mimic the conditions bacteria face during human infection. We investigated the expression of *sod* in *A. actinomycetemcomitans* strains in a bacteriostatic state induced by iron-chelator treatment and found that the expression pattern under iron-restricted conditions was similar to that observed in control conditions (Fig. S3). These results suggested *sod* regulation by ArcA likely occurs *in vivo* as well.

Bacterial resistance to ROS produced by the host immune system, such as macrophages, contributes to pathogen survival during host colonization (63). We investigated the survival of *A. actinomycetemcomitans* and its mutant strains in THP-1 macrophages and found that survival rates were increased in Δ*arcA* (Fig. 5A) but decreased in Δ*sod* (Fig. 5B). *A. actinomycetemcomitans* produces leukotoxin (LtxA) that induces cell death in macrophages (64). We analyzed the expression level of *ltxA* in Δ*arcA* by qRT-PCR and found that the mutant did not show increased expression of *ltxA* compared to WT (Fig. S5). These results suggest that ArcA activation in macrophages negatively affects bacterial survival through the regulation of *sod* expression.

Δ*arcB* presented similar phenotypic trends compared to those of Δ*arcA*, although some differences did not reach statistical significance (Fig. 1A and 3B, Fig. 4A and 5A; Fig. S3). We investigated the relationship between ArcA and ArcB in *A. actinomycetemcomitans*. We found that *arcB* inactivation resulted in a 2.31-fold increase in *arcA* expression, as determined by qRT-PCR analyses (Fig. 1B). Immunoblot analysis also revealed increased ArcA levels in Δ*arcB* cell lysates (Fig. 6B). Additionally, ArcA phosphorylation levels were reduced in Δ*arcB*; however, phosphorylated ArcA was not completely absent (Fig. 6C). These data suggest that ArcA expression is negatively regulated by phosphorylated ArcB and that ArcA is partially phosphorylated by ArcB in response to unknown stimuli. Furthermore, other factors are likely involved in ArcA phosphorylation. Therefore, the inactivated mutant of *arcB* was considered to present moderate phenotypes compared to those of Δ*arcA*. Yamamoto et al. reported potential crosstalk between non-cognate HKs and RRs in *E. coli*, such as RR CheY being phosphorylated by HK BaeS and HK DcuS or RR NarL being phosphorylated by HK BarA and HK UhpB (42). Wang et al. reported crosstalk between orphan RR SCO3818 and non-cognate HK SCO0203 in *Streptomyces coelicolor* (65). Yamamoto et al*.* reported that ArcB was the only HK capable of phosphorylating ArcA *in vivo*; however, they suggested potential crosstalk with other kinases *in vivo* (42). In this study, we tested whether two other TCSs were involved in ArcA phosphorylation using strain IDH781. QseBC, the most extensively studied TCS, was not investigated here because it is annotated as a pseudogene in the genome sequence of *A. actinomycetemcomitans* NUM4039. We found that the ArcA phosphorylation levels were not significantly reduced by these mutations. Therefore, these results suggest the involvement of additional kinases in ArcA phosphorylation in *A. actinomycetemcomitans*.

This study demonstrated that ArcA receives phosphate groups, in part from ArcB, and regulates the expression of genes, including *sod*, thereby contributing to the modulation of stress responses to H_2_O_2_ and O_2_^•−^. ArcAB is known to function as an oxygen sensor, typically acting under oxygen-limiting conditions. *A. actinomycetemcomitans* is a facultative anaerobe that can adapt to both aerobic and anaerobic conditions in the oral cavity. This bacterium may repress *sod* expression under anaerobic conditions to conserve energy.

## MATERIALS AND METHODS

### Bacterial strains and culture conditions

The bacterial strains used in this study are listed in Table 1. *A. actinomycetemcomitans* strains were cultured in AAGM. AAGM consists of 2.5% TSB (Becton, Dickinson and Company, Franklin Lakes, NJ, USA), 0.6% yeast extract (Nacalai Tesque, Kyoto, Japan), 1% glucose (FUJIFILM Wako Pure Chemical Corporation [hereafter FUJIFILM Wako], Osaka, Japan), and 0.4% sodium bicarbonate (Nacalai Tesque). *A. actinomycetemcomitans* strains were cultured at 37°C under ambient air (78% N_2_, 21% O_2_, 0.9% Ar, 0.04% CO_2_, and other gases) supplemented with 5% CO_2_ using a CO_2_ incubator. Spectinomycin (50 µg/mL) was added to the medium for culturing *A. actinomycetemcomitans* knockout strains. For complemented strains of *A. actinomycetemcomitans*, both spectinomycin (50 µg/mL) and chloramphenicol (1 µg/mL) were added to the medium.

**TABLE 1:** Bacterial strains and plasmids used in this study^*a*^

Bacterial strains/plasmids	Characteristics	Sources
*Aggregatibacter actinomycetemcomitans*		
NUM4039	Clinical isolate (serotype g)	(5)
Δ*arcA*	*arcA* mutant of NUM4039, Spec^r^	This study
Δ*arcB*	*arcB* mutant of NUM4039, Spec^r^	This study
Δ*sod*	*sod* mutant of NUM4039, Spec^r^	This study
*arcA* compl.	Complementation of Δ*arcA*, Spec^r^, Cp^r^	This study
*arcB* compl.	Complementation of Δ*arcB*, Spec^r^, Cp^r^	This study
*sod* compl.	Complementation of Δ*sod*, Spec^r^, Cp^r^	This study
*Streptococcus sanguinis*		
SK36	Clinical isolate	(50)
Δ*spxB*	*spxB* mutant of SK36	(51)
*Escherichia coli*		
XL10-Gold	Cloning host for recombinant plasmid	Stratagene
HB101-pRK2013	Mobilization helper strain	ATCC
HB101-pRK2013-pJAK16::*arcA*	Donor strain of *arcA*-pJAK16, Km^r^, Cp^r^	This study
HB101-pRK2013-pJAK16::*arcB*	Donor strain of *arcB*-pJAK16, Km^r^, Cp^r^	This study
HB101-pRK2013-pJAK16::*sod*	Donor strain of *sod*-pJAK16, Km^r^, Cp^r^	This study
BL21(DE3)/pREP4	Cloning host for recombinant plasmid, *lacI^q^*, Km^r^	This study
BL21(DE3)/pREP4-pQE30::*arcA*	BL21(DE3)/pREP4 carrying pQE30-*arcA*, Km^r^, Amp^r^	This study
Plasmid		
pJAK16	Broad host range plasmid	(45)
pJAK16::*arcA*	Complementation plasmid for *arcA*	This study
pJAK16::*arcB*	Complementation plasmid for *arcB*	This study
pJAK16::*sod*	Complementation plasmid for *sod*	This study
pRK2013	Helper plasmid for mobilization	(66)
pQE30	Expression plasmid for recombinant protein	Qiagen
pQE30::*arcA*	pQE30 containing *arcA*	This study

^*a*^ Spec^r^, spectinomycin resistance; Cp^r^, chloramphenicol resistance; Km^r^, kanamycin resistance; Amp^r^ ampicillin resistance.

*S. sanguinis* was cultured in TSB at 37°C under ambient air supplemented with 5% CO_2_. *E. coli* strains were cultured under ambient air in Luria–Bertani (LB) broth (Nacalai Tesque) with shaking (120 rpm). Ampicillin (100 µg/mL) and kanamycin (25 µg/mL) were added to the medium to maintain the pQE30 (Qiagen, Hilden, Germany) and pRK2013 plasmids, respectively.

All antibiotics were purchased from FUJIFILM Wako.

### Construction of knockout strains

Knockout strains of *arcA*, *arcB*, or *sod* were created by substituting each target gene with a spectinomycin resistance gene (*aad*9) derived from *Enterococcus faecalis* (67). In brief, three DNA fragments were amplified by PCR with KOD plus DNA polymerase (TOYOBO, Osaka, Japan): upstream and downstream regions flanking the target gene and the *aad*9 gene. These fragments were then fused by overlap extension PCR (68). The fused DNA fragment was separated by agarose gel electrophoresis, purified from the gel, and used for transformation.

*A. actinomycetemcomitans* was cultured overnight on TSB agar containing 5% heat-inactivated horse serum (Thermo Fisher Scientific, Waltham, MA, USA) (STSB) at 37°C in a CO_2_ incubator (5% CO_2_). The cells were resuspended in phosphate-buffered saline (PBS) to a concentration of approximately 10^10^ colony-forming units (CFUs)/mL. A 50 µL aliquot of the suspension was spotted onto prewarmed (37°C) STSB agar and incubated at 37°C in a CO_2_ incubator (5% CO_2_) for 2 h. The fused DNA fragment (200 ng) was mixed with the cells using a sterile loop. The plate was incubated at 37°C in a CO_2_ incubator (5% CO_2_) for 8 h. The cells were scraped from the plate and resuspended in 1 mL of TSB containing 0.6% yeast extract (TSB-YE). Serial dilutions were plated on TSB-YE agar containing spectinomycin (50 µg/mL). The plates were incubated at 37°C in a CO_2_ incubator (5% CO_2_) for 4 days. Genomic DNA was extracted from the resulting colonies, and gene inactivation was confirmed by colony PCR. The primers used in this study are listed in Table S4.

### Construction of complemented strains

For the construction of *A. actinomycetemcomitans* complemented strains, the broad-host-range plasmid pJAK16 (45) was used. pJAK16 can be mobilized from *E. coli* to other Gram-negative hosts by the conjugative IncP helper plasmid pRK2013 (66). DNA fragments containing either the *arcA*, *arcB*, or *sod* structural gene with its promoter region and a fragment of pJAK16 were amplified using PrimeSTAR Max DNA Polymerase (Takara Bio, Kusatsu, Japan). These DNA fragments were fused using In-Fusion Snap Assembly Master Mix (Takara Bio), and the resulting plasmids were subsequently transformed into *E. coli* HB101 harboring pRK2013. The *E. coli* transformants (HB101-pRK2013-pJAK16::*arcA*, HB101-pRK2013-pJAK16::*arcB*, and HB101-pRK2013-pJAK16::*sod*) were used as donor strains for conjugation. The donor *E. coli* strains were cultured overnight in LB broth at 37°C, and the recipient *A. actinomycetemcomitans* mutant strains were cultured at 37°C in a CO_2_ incubator (5% CO_2_) for 12 h in AAGM broth. These cultures were mixed at a volume ratio of 1:99 (*E. coli: A. actinomycetemcomitans*) and incubated on TSB-YE agar plates at 37°C in a CO_2_ incubator (5% CO_2_) for 16 h. The resulting colonies were scraped from the plates and resuspended in 1 mL of TSB-YE broth. Serial dilutions were plated on TSB-YE agar containing spectinomycin (50 µg/mL) and chloramphenicol (1 µg/mL). Plates were incubated at 37°C in a CO_2_ incubator (5% CO_2_) for 4 days. Genomic DNA was extracted from the resulting colonies, and gene complementation was confirmed by PCR. The primers used in this study are listed in Table S4.

### Growth kinetics

A total of 10^8^ CFUs of *A. actinomycetemcomitans* strain NUM4039 (WT) and its mutant strains were grown in 10 mL of AAGM broth at 37°C under ambient air supplemented with 5% CO_2_. The OD_660_ was monitored for 24 h. For bacteriostatic conditions, 2,2′-bipyridyl (FUJIFILM Wako) was used as an iron chelator. The *A. actinomycetemcomitans* strains were grown until the OD_660_ reached 0.2 (early exponential phase) and then treated with 8 mM 2,2′-bipyridyl. As the vehicle control, the same amount of ethanol (FUJIFILM Wako) was used.

### RNA-seq analysis

A total of 10^8^ CFUs of *A. actinomycetemcomitans* NUM4039 (WT) and its Δ*arcA* mutant strain were grown in 10 mL of AAGM broth at 37°C under ambient air supplemented with 5% CO_2_ until the OD_660_ reached 0.6 (mid-exponential phase). Total RNA was extracted using Isogen II (NIPPON GENE, Tokyo, Japan) according to the manufacturer’s instructions. RNA-seq analysis was performed by Rhelixa Inc. (Tokyo, Japan) using GCF_001547775.1 as the reference genome. The FPKM values were calculated, and the values below 10 were excluded from analysis. Differential gene expression was represented as FPKM ratios (Δ*arcA*/WT).

### qRT-PCR

A total of 10^8^ CFUs of *A. actinomycetemcomitans* strain NUM4039 (WT) and its mutant strains were grown in 10 mL of AAGM broth at 37°C under ambient air supplemented with 5% CO_2_. Total RNA was extracted and treated with deoxyribonuclease (RT Grade) for Heat Stop (NIPPON GENE) to remove contaminating genomic DNA. Complementary DNA (cDNA) was synthesized using a Transcriptor First Strand cDNA Synthesis Kit (Roche Diagnostics, Basel, Switzerland). qRT-PCR was performed using FastStart Essential DNA Green Master reaction mix on a LightCycler 96 instrument (Roche Diagnostics). The _∆∆_*C*_T_ method was used to determine the relative transcript levels. The *C*_T_ value of *gapdh* served as an internal control. The primers used in this study are listed in Table S4.

### Superoxide killing assay

A total of 5 × 10^7^ CFUs of *A. actinomycetemcomitans* strains were grown in 5 mL of AAGM broth at 37°C under ambient air supplemented with 5% CO_2_ until the OD_660_ reached 0.6. The cells equivalent to 10^8^ CFUs were suspended in 1 mL of AAGM containing 250 µM xanthine and 0.1 U of xanthine oxidase (Roche Diagnostics). The suspension was incubated at 37˚C for 30 min. Serial dilutions were plated on TSB-YE agar plates and incubated at 37°C in a CO_2_ incubator (5% CO_2_) for 3 days. Colonies were counted, and survival rates were calculated. CFU counts from control samples without xanthine and xanthine oxidase were set as 100%.

### H_2_O_2_ susceptibility test

A total of 5 × 10^7^ CFUs of *A. actinomycetemcomitans* strains were cultured at 37°C in 5 mL of AAGM broth at 37°C under ambient air supplemented with 5% CO_2_ until the OD_660_ reached 0.6. The cells equivalent to 10^8^ CFUs were suspended in 5 mL of prewarmed TSB-YE medium containing 0.75% agar, and the suspension was poured over TSB-YE agar plates. Following solidification, sterile paper discs (Toyo Roshi, Tokyo, Japan) containing 10 µL of 3% H_2_O_2_ (FUJIFILM Wako) were placed on the agar and incubated at 37°C in a CO_2_ incubator (5% CO_2_) for 2 days. Growth inhibition was analyzed by measuring the distance from the edges of the inhibition zones to the edges of the paper discs in three directions.

### H_2_O_2_ susceptibility test with *S. sanguinis*

A total of 5 × 10^7^ CFUs of *S. sanguinis* strain SK36 and its isogenic *spxB* mutant (lacking pyruvate oxidase) were grown overnight in 5 mL of TSB at 37°C under ambient air supplemented with 5% CO_2_ until the OD_660_ reached 0.8. The cells were adjusted to 10^8^ CFUs/mL in TSB. Ten microliters of the suspension was spotted onto TSB agar plates and incubated at 37°C for 12 h. *A. actinomycetemcomitans* strains (indicator bacteria) were cultured in AAGM broth at 37°C under ambient air supplemented with 5% CO_2_ until the OD_660_ reached 0.6. The cells equivalent to 10^8^ CFUs were suspended in 5 mL of prewarmed TSB-YE medium containing 0.75% agar, and this suspension was poured over *S. sanguinis* colonies growing on TSB agar. After solidification, the plates were incubated at 37°C in a CO_2_ incubator (5% CO_2_) for 2 days. Growth inhibition was analyzed by measuring the distance from the edges of the inhibition zone to the edges of *S. sanguinis* colonies in three directions.

### Evaluation of bacterial survival in THP-1 macrophages

The human monocytic cell line THP-1 was obtained from RIKEN Bio Resource Center (RCB1189, RIKEN BRC, Ibaraki, Japan). The cells were maintained in RPMI 1640 medium (FUJIFILM Wako) supplemented with 10% fetal bovine serum (FBS) (Thermo Fisher Scientific), 100 U/mL potassium penicillin G, and 0.1 mg/mL streptomycin sulfate at 37°C in a CO_2_ incubator (5% CO_2_). THP-1 cells were seeded into 24-well plates at 2 × 10^5^ cells per well. After 48 h, the cells were treated with 25 ng/mL phorbol 12-myristate 13-acetate (PMA) (Sigma-Aldrich, St. Louis, MO, USA) and incubated for 3 days to induce macrophage differentiation. The medium was then replaced with antibiotic-free RPMI 1640 containing 10% FBS 24 h before infection.

*A. actinomycetemcomitans* strains were grown in AAGM broth at 37°C under ambient air supplemented with 5% CO_2_ until the OD_660_ reached 0.6. For opsonization, the cells equivalent to 10^8^ CFUs were incubated in AAGM broth containing 20% pooled human serum (Funakoshi Co., Ltd., Tokyo, Japan) at 37°C for 30 min. The initial bacterial CFUs were determined by plating serial dilutions on TSB-YE agar and incubating at 37°C in a CO_2_ incubator (5% CO_2_) for 3 days.

Bacterial suspensions (10^7^ CFUs per well) were added to the macrophages at a multiplicity of infection (MOI) of 50 and incubated at 37°C in a CO_2_ incubator (5% CO_2_) for 90 min. After three washes with PBS, the extracellular bacteria were killed by treating them with 100 µg/mL gentamycin (FUJIFILM Wako) in RPMI at 37°C for 15 min. After three washes with PBS, the macrophages were lysed with 1% saponin (Nacalai Tesque) at 37°C for 15 min. To recover the viable bacteria, serial dilutions of the cell lysates were plated on TSB-YE agar and incubated at 37°C in a CO_2_ incubator (5% CO_2_) for 4 days. Colonies were counted, and bacterial survival was calculated as the percentage of recovered CFUs relative to the initial inoculum.

### Preparation of recombinant ArcA and antiserum

To construct His-tagged recombinant ArcA, the *arcA* gene was cloned and inserted into pQE30 (Qiagen) using In-Fusion Snap Assembly Master Mix (Takara Bio). The resulting plasmid pQE30::*arcA* was transformed into *E. coli* XL10-Gold and purified with a Quantum Prep Plasmid Kit (Bio-Rad Laboratories, Inc., Hercules, CA, USA). For protein purification, *E. coli* BL21 (DE3) transformed with both pREP4 (Qiagen) and pQE30::*arcA* was grown in LB broth supplemented with 100 µg/mL ampicillin and 25 µg/mL kanamycin at 37°C with shaking at 140 rpm until the OD_660_ reached 0.2. Protein expression was induced with 0.1 mM IPTG at 37°C for 5 h. His-tagged ArcA was purified by Ni-NTA affinity chromatography according to the manufacturer’s instructions (Qiagen). Rabbit antiserum against His-tagged ArcA was obtained from Scrum Inc. (Tokyo, Japan). The primers used in this study are listed in Table S4.

### Immunoblotting

A total of 10^8^ CFUs of *A. actinomycetemcomitans* strains were grown in 10 mL of AAGM broth at 37°C under ambient air supplemented with 5% CO_2_ until the OD_660_ reached 0.6. The bacterial cultures were subsequently centrifuged at 3,000 × *g* for 10 min, after which the cell pellets were resuspended in 500 µL of ice-cold PBS. The suspension was transferred to a 2 mL tube containing 200 mg of glass beads (100 µm diameter) (Tommy Seiko Co., Ltd., Tokyo, Japan) and disrupted using a Micro Smash (Tommy Seiko Co., Ltd.) at 3,500 rpm for 30 s. Samples were centrifuged at 12,000 × *g* for 5 min. Supernatants were collected and used as whole-cell extracts. Proteins from 5 µL aliquots were separated on either 12% polyacrylamide gels or 12.5% SuperSep Phos-tag precast gels (FUJIFILM Wako). After electrophoresis, the Phos-tag gels were washed three times with 5 mM EDTA (Dojindo Laboratories Co., Ltd., Kumamoto, Japan) for 10 min each to remove zinc ions. The separated proteins were transferred to Whatman Protran nitrocellulose membranes (Cytiva, Marlborough, MA, USA) and blocked overnight at 4°C in TBS-T containing 2% skim milk (FUJIFILM Wako). The membranes were incubated for 1 h with ArcA antiserum (1:20,000) in TBS-T containing 1% skim milk. After being washed with TBS-T, the membranes were incubated for 1 h with horseradish peroxidase-conjugated anti-rabbit IgG antibody (Promega, Madison, WI, USA) (1:2,500) in TBS-T. The membranes were washed five times with TBS-T and treated with Western Lightning ECL Pro reagent (PerkinElmer, Waltham, MA, USA). ArcA signals were captured at 60 images per min using a Bio-Rad ChemiDoc MP imaging system (Bio-Rad Laboratories, Inc.) until signal saturation. ArcA levels were quantified by measuring the signal intensity from images captured immediately before saturation using Image Lab 6.0.1 (Bio-Rad Laboratories, Inc.). To quantify the phosphorylation levels of ArcA, the intensities of phosphorylated and unphosphorylated ArcA were measured from images obtained using SuperSep Phos-tag precast gels. The phosphorylation levels of ArcA was calculated using the following formula: phosphorylated ArcA intensity/(unphosphorylated ArcA intensity + phosphorylated ArcA intensity) × 100.

### Statistical analysis

Statistical analyses were performed using EZR (version 1.27) (69), a graphical user interface for R (R Foundation for Statistical Computing, Vienna, Austria). Significant differences were determined by one-way analysis of variance followed by Tukey’s multiple-comparison test. Differences were considered statistically significant at *P* < 0.05.

## Data Availability

RNA-seq data are available in the Gene Expression Omnibus under accession number GSE284428.

## AFTER EPUB

*[This article was published on 16 April 2025 with errors in the introduction and references. The errors were corrected in the current version, posted on 23 April 2025.]*

## SUPPLEMENTAL MATERIAL

Supplemental figures msphere.00019-25-s0001.pdf Figures S1 to S5.

Supplemental tables msphere.00019-25-s0002.pdf Tables S1 to S4.

ASM does not own the copyrights to Supplemental Material that may be linked to, or accessed through, an article. The authors have granted ASM a non-exclusive, world-wide license to publish the Supplemental Material files. Please contact the corresponding author directly for reuse.
