# Supplementary material for: The ArcAB two-component system is associated with the susceptibility of Aggregatibacter actinomycetemcomitans to superoxide and hydrogen peroxide
Source: mSphere. 2025 Apr 16;10(5):e00019-25. doi: 10.1128/msphere.00019-25 (PMC12108069; doi:10.1128/msphere.00019-25)
Supplement: Supplemental figures — Figures S1 to S5. [file msphere.00019-25-s0001.pdf]

Supplemental Figure 1.

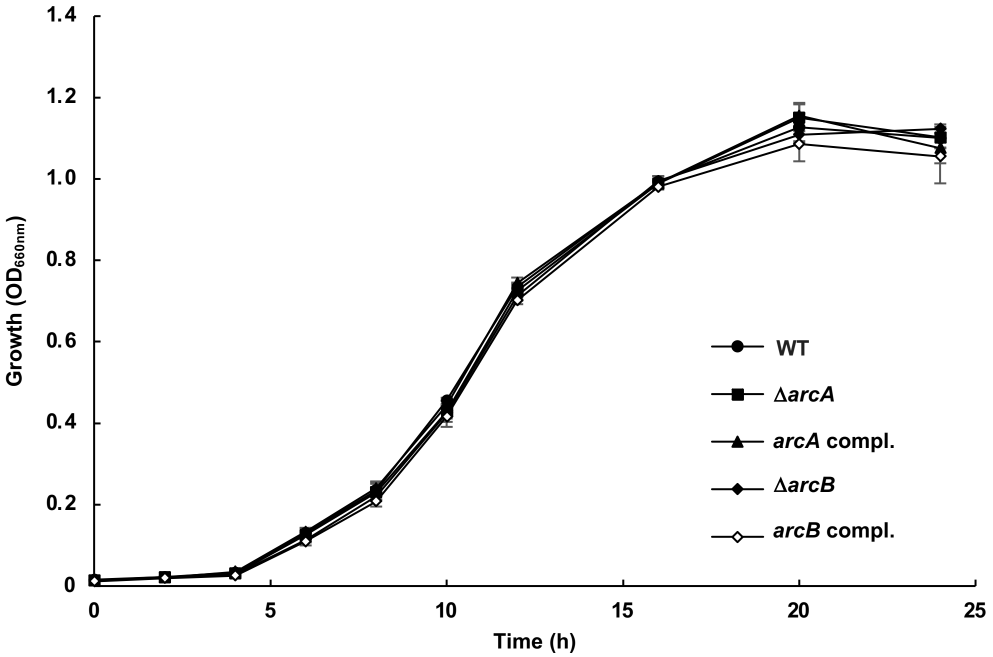

Supplemental Fig. 1. Growth kinetics of *A. actinomycetemcomitans* strains.

The *A. actinomycetemcomitans* NUM4039 wild-type strain (WT), *arcA* and *arcB* mutants (*ΔarcA* and *ΔarcB*), and their respective complemented strains (*arcA* compl. and *arcB* compl.) were grown in AAGM broth at 37˚C under ambient air supplemented with 5% CO_2. The OD_{660} was recorded for 24 h. The data represent the means ± SDs from three independent experiments, each with three biological replicates.

Supplemental Figure 2.

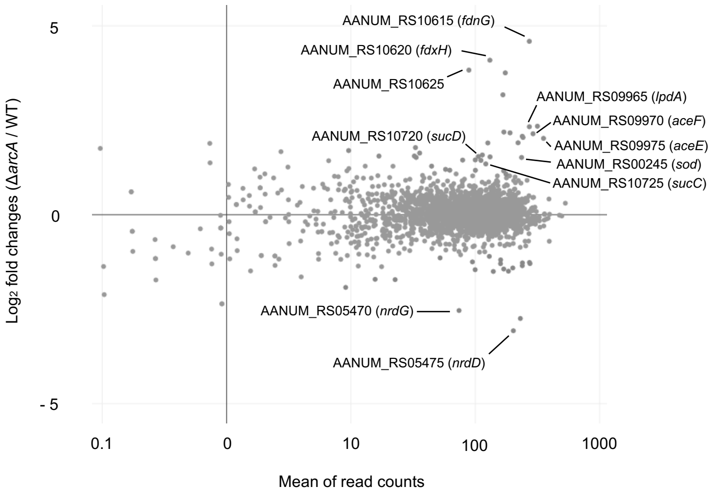

Supplemental Fig. 2. MA plot visualizing the differentially expressed genes between WT and Δ*arcA*.

The differentially expressed genes were analyzed in cells grown to the mid-exponential phase ($OD_{660} = 0.6$) by RNA-seq. The MA plot shows the $\log_2$ fold changes of gene expression in the NUM4039 *ΔarcA* compared to the WT and the mean of read counts. The plots showing *sod* and the genes involved in carbon source metabolism and formate dehydrogenase were labeled. The MA plot was generated by plotly (version 4.9.2.1).

Supplemental Figure 3.

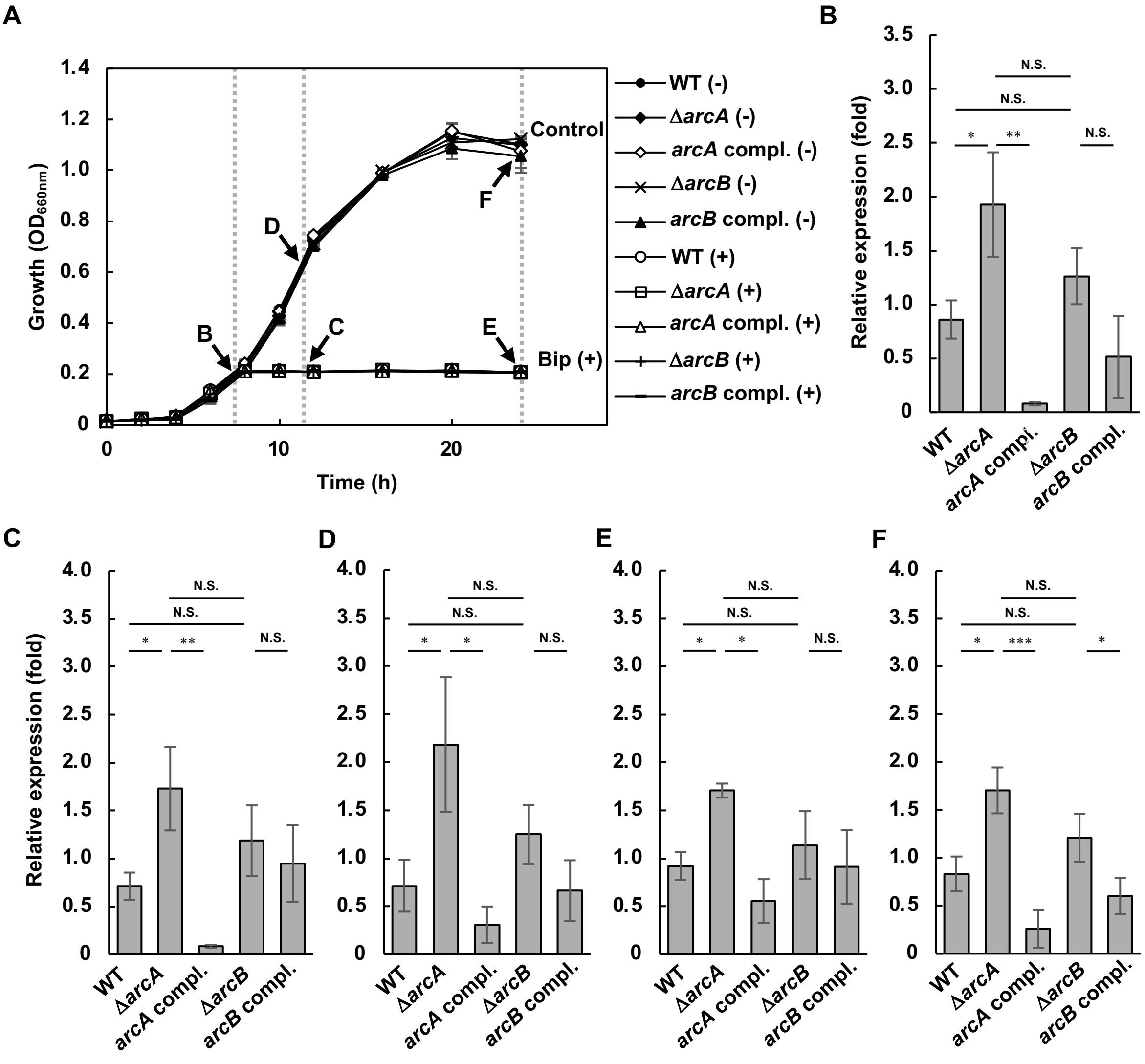

Supplemental Fig. 3. Growth kinetics and *sod* expression in *A. actinomycetemcomitans* strains under iron-restricted conditions.

(A) The growth kinetics when the *A. actinomycetemcomitans* strain NUM4039 and its isogenic mutants were treated with 8 mM 2,2'-bipyridyl (Bip) or vehicle at early-exponential phase ($OD_{660} = 0.2$). The arrows with alphabet in panel A indicate the timing of sampling and correspond to panels B - F. The *sod* expression levels of *A. actinomycetemcomitans* NUM4039 strains were determined in early-exponential phase cells (B), after treatment with Bip (C) or vehicle (D) for 4 h, and stationary phase cells (24 h cultivation) treated with Bip (E) or vehicle (F). The expression levels were normalized with *gapdh* serving as an internal control. The relative expression (fold) was determined using the expression level in mid-exponential phase ($OD_{660} = 0.6$) cells of WT as the calibrator. The data represent the means ± SDs from three independent experiments, each with three biological replicates. Statistical significance was determined by Tukey's test (*, $p < 0.05$; **, $p < 0.001$; and ***, $p < 0.001$; and N.S., not significant).

Supplemental Figure 4.

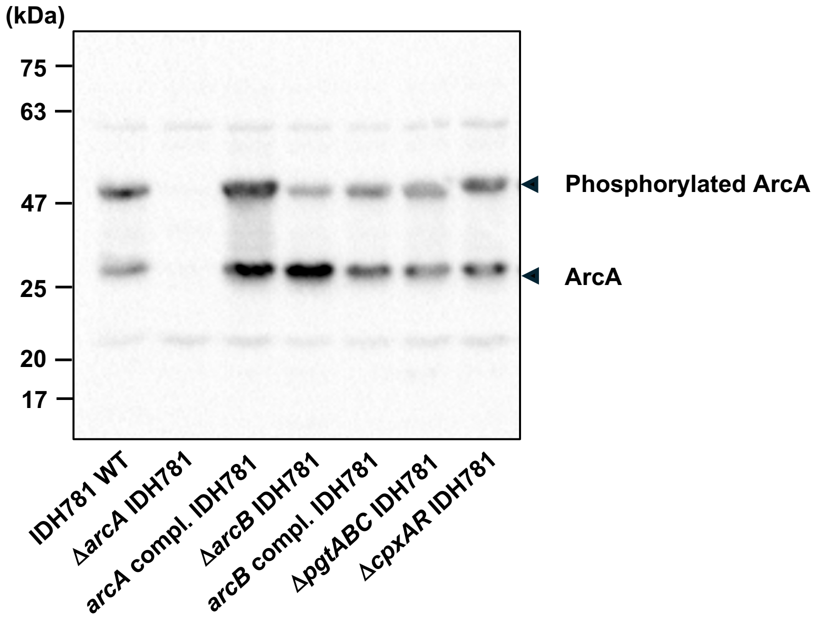

Supplemental Fig. 4. Evaluation of ArcA phosphorylation levels in the *A. actinomycetemcomitans* strain IDH781 and its mutants.

Total proteins in homogenates of the *A. actinomycetemcomitans* IDH781 WT and its mutant strains grown to mid-exponential phase (OD_{660} = 0.6) were sedimented in a 12.5% Phos-tag gel. Immunoblot analysis was conducted with rabbit anti-ArcA antiserum and horseradish peroxidase-conjugated anti-rabbit IgG antibody. A representative picture of 3 independent tests is shown.

Supplemental Figure 5.

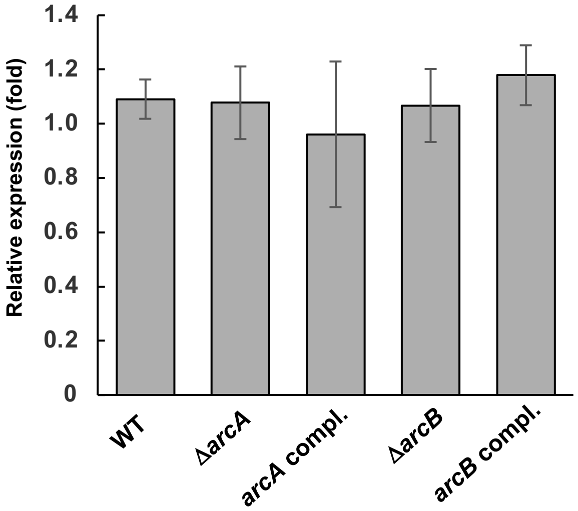

Supplemental Fig. 5. *ltxA* expression in *A. actinomycetemcomitans* strains.

The *ltxA* expression levels of the *A. actinomycetemcomitans* strain NUM4039 and its isogenic mutants were determined in cells grown to the mid-exponential phase ($OD_{660} = 0.6$), with *gapdh* serving as an internal control. The relative expression (fold) was determined using the expression level in WT as the calibrator. The data represent the means ± SDs from three independent experiments, each with three biological replicates. Statistical significance was not detected among tested strains by Tukey's test.
