# Supplementary material for: The ArcAB two-component system is associated with the susceptibility of Aggregatibacter actinomycetemcomitans to superoxide and hydrogen peroxide
Source: mSphere. 2025 Apr 16;10(5):e00019-25. doi: 10.1128/msphere.00019-25 (PMC12108069; doi:10.1128/msphere.00019-25)
Supplement: Supplemental tables — Tables S1 to S4. [file msphere.00019-25-s0002.pdf]

Supplemental Table 1. Upregulated genes in Δ*arcA* compared to WT (> 2-fold).

Locus tag	Gene	Products	FPKM		Fold change
			WT	Δ*arcA*	
AANUM_RS10615	*fdnG*	Formate dehydrogenase-N subunit alpha	69.34	1692.03	24.40
AANUM_RS10620	*fdxH*	Formate dehydrogenase subunit beta	41.80	684.91	16.38
AANUM_RS10625		Formate dehydrogenase subunit gamma	24.23	360.44	14.88
AANUM_RS07095		NAD(P)/FAD-dependent oxidoreductase	67.56	932.56	13.80
AANUM_RS02040		DASS family sodium-coupled anion symporter	64.45	756.85	11.75
AANUM_RS09965	*lpdA*	Dihydrolipoyl dehydrogenase	588.21	2987.38	5.08
AANUM_RS03760		tRNA-Phe	21.71	109.10	5.03
AANUM_RS06900	*lldD*	FMN-dependent L-lactate dehydrogenase LldD	1346.67	6661.98	4.94
AANUM_RS01725	*lipA*	Lipoyl synthase	254.28	1185.11	4.66
AANUM_RS10730	*odhB*	2-oxoglutarate dehydrogenase complex dihydrolipoyllysine-residue succinyltransferase	236.62	1064.42	4.50
AANUM_RS09970	*aceF*	Pyruvate dehydrogenase complex dihydrolipoyllysine-residue acetyltransferase	752.82	3366.98	4.47
AANUM_RS06895		L-lactate permease	406.21	1763.26	4.34
AANUM_RS10735	*sucA*	2-oxoglutarate dehydrogenase E1 component	251.51	1018.92	4.05
AANUM_RS09975	*aceE*	Pyruvate dehydrogenase (acetyl-transferring), homodimeric type	1086.80	4265.07	3.92
AANUM_RS12340		Hypothetical protein	151.67	539.22	3.56
AANUM_RS08260		DoxX family protein	1462.45	5187.45	3.55
AANUM_RS08660	*ftnA*	Non-heme ferritin	324.06	1100.42	3.40
AANUM_RS06355		Hypothetical protein	18.75	53.84	2.87
AANUM_RS00245	*sod*	Superoxide dismutase	1307.36	3714.86	2.84
AANUM_RS08305		TOBE domain-containing protein	30.88	85.12	2.76
AANUM_RS00465		Helix-hairpin-helix domain-containing protein	13.87	38.03	2.74
AANUM_RS10720	*sucD*	Succinate--CoA ligase subunit alpha	113.07	309.13	2.73
AANUM_RS12030		Integrase core domain-containing protein	15.86	43.18	2.72
AANUM_RS06345		tRNA-Lys	62.41	168.19	2.69
AANUM_RS07460		tRNA-Arg	21.43	51.59	2.41
AANUM_RS10725	*sucC*	ADP-forming succinate--CoA ligase subunit beta	127.23	304.76	2.40
AANUM_RS13225		Hypothetical protein	77.69	179.83	2.31
AANUM_RS12020		Dethiobiotin synthase	191.19	426.44	2.23
AANUM_RS00750		Ribosome alternative rescue factor ArfA	18.28	40.43	2.21
AANUM_RS07040		Hypothetical protein	15.62	34.02	2.18

AANUM_RS08920	*cspD*	Cold shock domain-containing protein CspD	1881.90	4096.39	2.18
AANUM_RS03355		tRNA-Tyr	1458.10	3121.41	2.14
AANUM_RS12930		DUF5363 domain-containing protein	189.67	401.98	2.12
AANUM_RS13135		Hypothetical protein	88.31	185.58	2.10
AANUM_RS07470		tRNA-Pro	48.21	100.95	2.09
AANUM_RS08380		ROK family protein	388.07	808.77	2.08
AANUM_RS00765		YdcH family protein	145.12	301.48	2.08
AANUM_RS09165	*cmk*	(d) CMP kinase	667.55	1374.96	2.06
AANUM_RS08655	*ftnA*	Non-heme ferritin	320.79	926.83	2.89
AANUM_RS08385	*bioD*	Dethiobiotin synthase	401.65	815.37	2.03

FPKM, fragments per kilobase of transcript per million fragments mapped.

Supplemental Table 2. Downregulated genes in Δ*arcA* compared to WT (< 0.5-fold).

Locus tag	Gene	Products	FPKM		Fold change
			WT	Δ*arcA*	
AANUM_RS05470	*nrdG*	Anaerobic ribonucleoside-triphosphate reductase-activating protein	362.78	22.29	0.06
AANUM_RS05475	*nrdD*	Anaerobic ribonucleoside-triphosphate reductase	871.20	58.15	0.06
AANUM_RS06225	*pgtP*	Phosphoglycerate transporter protein PgtP	2102.70	295.82	0.14
AANUM_RS10645		Respiratory chain complex I subunit 1 family protein	656.26	224.55	0.34
AANUM_RS10640	*hyfB*	Hydrogenase 4 subunit B	631.54	216.28	0.34
AANUM_RS10675		NADH-quinone oxidoreductase subunit B family protein	913.54	337.43	0.35
AANUM_RS10650		Hydrogenase 4 subunit D	1083.13	392.63	0.36
AANUM_RS04475		IS30 family transposase	27.67	10.16	0.36
AANUM_RS10685	*hycI*	Hydrogenase maturation peptidase HycI	484.78	180.26	0.37
AANUM_RS00810	*rrf*	5S ribosomal RNA	32.00	11.91	0.37
AANUM_RS10670		Formate hydrogenlyase complex iron-sulfur subunit	1419.90	536.41	0.38
AANUM_RS10665		Hydrogenase large subunit	1588.40	601.77	0.38
AANUM_RS10680		Formate hydrogenlyase maturation HycH family protein	578.14	222.15	0.38
AANUM_RS05410		tRNA-Glu	170.95	65.91	0.38
AANUM_RS11635	*grcA*	Autonomous glycyl radical cofactor GrcA	10926.50	4266.21	0.39
AANUM_RS09600		ATP-binding cassette domain-containing protein	37.64	15.08	0.40
AANUM_RS00945		Cof-type HAD-IIB family hydrolase	55.56	22.38	0.40
AANUM_RS10660		Hydrogenase 4 subunit F	1697.40	686.10	0.40
AANUM_RS02075		UDP-glucose 6-dehydrogenase	32.17	13.05	0.41
AANUM_RS10655	*hyfE*	Hydrogenase 4 membrane subunit	7353.90	2988.00	0.41
AANUM_RS06220		TonB-dependent receptor	384.95	159.62	0.41
AANUM_RS00950	*manZ*	PTS mannose transporter subunit IID	144.16	60.89	0.42
AANUM_RS10485		Acyltransferase family protein	37.85	16.45	0.43
AANUM_RS12545	*yidD*	Membrane protein insertion efficiency factor YidD	438.52	199.21	0.45
AANUM_RS10635		4Fe-4S dicluster domain-containing protein	760.29	351.25	0.46

AANUM_RS08200		DUF302 domain-containing protein	578.18	267.40	0.46
AANUM_RS11765		Mannose/ fructose/ sorbose PTS transporter subunit IIB	126.99	62.24	0.49
AANUM_RS09470	*metE*	5-methyltetrahydropteroyltriglutamate--homocysteine S-methyltransferase	1218.20	608.52	0.49

FPKM, fragments per kilobase of transcript per million fragments mapped.

Supplemental Table 3. Strains used in supplemental figure 4.

Bacterial Strains	Characteristics	Sources
Aggregatibacter actinomycetemcomitans		
IDH781	Clinical isolated (serotype d)	(1)
Δ*arcA* IDH781	*arcA* mutant of IDH781, $Spec^r$	This study
Δ*arcB* IDH781	*arcB* mutant of IDH781, $Spec^r$	This study
Δ*cpxAR* IDH781	*cpxA* and *cpxR* mutant of IDH781, $Spec^r$	This study
Δ*pgtABC* IDH781	*pgtA*, *pgtB* and *pgtC* mutant of IDH781, $Spec^r$	This study
arcA compl. IDH781	Complementation of Δ*arcA* IDH781, $Spec^r$, Cp^r	This study
arcB compl. IDH781	Complementation of Δ*arcB* IDH781; $Spec^r$, Cp^r	This study

(1) May AC *et al*. 2016. Complete Genome Sequence of *Aggregatibacter actinomycetemcomitans* Strain IDH781. Genome Announc 4.

Supplemental Table 4. Primers used in this study.

Primer	Sequence (5′ – 3′)
For mutant construction	
Upstream region of *arc*A	F: CCCTGAATTTTTACAGGTGC
	R: ATGTATTCAATGGCAAATCCTTATTTTGTC
aad9 for substitution to *arcA*	F: GGATTTGCCATTGAATACATAC
	R: TGATTTTTCTTTATAATTTTTTTAATCTG
Downstream region of *arc*A	F: AAAATTATAAAGAAAAATCAAAGAGAAATCAGC
	R: GGATATGGACGAAGAACATTTG
Upstream region of *arcB*	F: GAAGTGGTTCCCGAATTTA
	R: ATGTATTCAAAGCATTTCGTGACAAGAG
aad9 for substitution to *arcB*	F: ACGAAATGCTTTGAATACATACGAACAAATTAATA
	R: CGTTTTTCGATTATAATTTTTTTAATC
Downstream region of *arcB*	F: AAAATTATAATCGAAAAACGGTTGGAAC
	R: AGTAACGGTAAAGTCGCTGG
Upstream region of *sod*	F: GTTTACAAAACCAACCTGTT
	R: ATGTATTCAAAACGTTTTCCTTTTCTTCG
aad9 for substitution to *sod*	F: GGAAAACGTTTTGAATACATACGAACAAATTAAT
	R: GTTTAGCAAATTATAATTTTTTTAATCTGTTATTTAAATA
Downstream region of *sod*	F: AAAATTATAATTTGCTAAACAGATAAATTCC
	R: TTTATTCTTTTTTACCTGCC
For complementation	
arcA for infusion with pJAK16	F: CCGGGGATCCATGTTGCAACTATAAAATAGATT
	R: ATGCCTGCAGTTAGGATTCCAAGTCACC
pJAK16 for infusion with *arcA*	F: GGAATCCTAACTGCAGGCAAGCT
	R: GTTGCAACATGATCCCCGGGTACCGAGCTC
arcB for infusion with pJAK16	F: CCGGGGATCCTCGAAATTCATTGTGTTTTT
	R: ATGCCTGCAGTCAATAACCTGCCAACCA
pJAK16 for infusion with *arcB*	F: AGGTTATTGACTGCAGGCATGCAAGCT
	R: TGAATTTCGAGGATCCCCGGGTACCGA
sod for infusion with pJAK16	F: CCGGGGATCCGCCGTATTATCAACATGGTGG
	R: ATGCCTGCAGTTAGTGTGGTGTACCGGC
pJAK16 for infusion with *sod*	F: ACCACACTAACTGCAGGCAGGCATGCAAGCT
	R: ATAATACGGCGGATCCCCGGGTACCGA
For recombinant protein	

arcA for infusion with pQE30	F: TCACCATCTCACCAAACACCACGAATTTTAAT
	R: TTGGCTGCAGGGATTCCAAGTCACCACAGA
pQE30 for infusion with *arcA*	F: GTGGTGTTTGGTGATGGTGATGGTGATG
	R: CTTGGAATCCCTGCAGCCAAGCTTAATT
For quantitative RT-PCR	
arcA	F: AACAGTCGCACGTTGGTTAC
	R: CGTGAATGGTGGCGATGATT
arcB	F: TTTTCTCCCGCCGCTTATTG
	R: GGATTCCGCTTGCGGTTTAT
sod	F: ACGTTCATTGGCGAAATCCC
	R: TCTACCGCCAACCAAGACAA
ltxA	F: ACCTGTCGCAGGGTTAATTG
	R: GCATCTGCGATCCCTGTATT
gapdh	F: CCCAAAACATCATCCCATCTTC
	R: GGAACACGGAACGCCATAC
